# A Prospective Investigation of Tumor Hypoxia Imaging with ^68^Ga-Nitroimidazole PET/CT in Patients with Carcinoma of the Cervix Uteri and Comparison with ^18^F-FDG PET/CT: Correlation with Immunohistochemistry

**DOI:** 10.3390/jcm11040962

**Published:** 2022-02-12

**Authors:** Kgomotso M. G. Mokoala, Ismaheel O. Lawal, Letjie C. Maserumule, Khanyisile N. Hlongwa, Honest Ndlovu, Janet Reed, Meshack Bida, Alex Maes, Christophe van de Wiele, Johncy Mahapane, Cindy Davis, Jae Min Jeong, Gbenga Popoola, Mariza Vorster, Mike M. Sathekge

**Affiliations:** 1 Department of Nuclear Medicine, University of Pretoria, Pretoria 0001, South Africa; kgomotso.mokoala@up.ac.za (K.M.G.M.); ismaheellawal@gmail.com (I.O.L.); letjie.maserumule@gmail.com (L.C.M.); khanyi29@gmail.com (K.N.H.); ndlovuhonest@gmail.com (H.N.); drjanreed@gmail.com (J.R.); alex.maes@azgroeninge.be (A.M.); cvdwiele@hotmail.com (C.v.d.W.); jkmahapane@gmail.com (J.M.); sbahtherapy@gmail.com (C.D.); marizavorster@gmail.com (M.V.); 2 Nuclear Medicine Research Infrastructure (NuMeRI), Steve Biko Academic Hospital, Pretoria 0001, South Africa; 3 Department of Anatomical Pathology, National Health Laboratory Services, Pretoria 0001, South Africa; meshack.bida@nhls.ac.za; 4 Department of Nuclear Medicine, Katholieke University Leuven, 8500 Kortrijk, Belgium; 5 Department of Radiology and Nuclear Medicine, University of Ghent, 9000 Ghent, Belgium; 6 Radiation Applied Life Sciences, Department of Nuclear Medicine, Institute of Radiation Medicine, Seoul National University College of Medicine, Seoul 03080, Korea; jmjng@snu.ac.kr; 7 Cancer Research Institute, Seoul National University College of Medicine, Seoul 03080, Korea; 8 Department of Epidemiology and Community Health, University of Ilorin, Ilorin 240102, Nigeria; g.popoola45@gmail.com

**Keywords:** cervical cancer, hypoxia, ^68^Ga-Nitroimidazole, ^18^F-FDG, HIF-1α, immunohistochemistry

## Abstract

Hypoxia in cervical cancer has been associated with a poor prognosis. Over the years ^68^Ga labelled nitroimidazoles have been studied and have shown improved kinetics. We present our initial experience of hypoxia Positron Emission Tomography (PET) imaging in cervical cancer with ^68^Ga-Nitroimidazole derivative and the correlation with ^18^F-FDG PET/CT and immunohistochemistry. Twenty women with cervical cancer underwent both ^18^F-FDG and ^68^Ga-Nitroimidazole PET/CT imaging. Dual-point imaging was performed for ^68^Ga-Nitroimidazole PET. Immunohistochemical analysis was performed with hypoxia inducible factor-1α (HIF-1α). We documented SUVmax, SUVmean of the primary lesions as well as tumor to muscle ratio (TMR), tumor to blood (TBR), metabolic tumor volume (MTV) and hypoxic tumor volume (HTV). There was no significant difference in the uptake of ^68^Ga-Nitroimidazole between early and delayed imaging. Twelve patients had uptake on ^68^Ga-Nitroimidazole PET. Ten patients demonstrated varying intensities of HIF-1α expression and six of these also had uptake on ^68^Ga-Nitroimidazole PET. We found a strong negative correlation between HTV and immunohistochemical staining (r = −0.660; *p* = 0.019). There was no correlation between uptake on PET imaging and immunohistochemical analysis with HIF-1α. Two-thirds of the patients demonstrated hypoxia on ^68^Ga-Nitroimidazole PET imaging.

## 1. Introduction

Despite widespread awareness programs and improvements in screening, cervical cancer continues to be a major cause of morbidity and mortality amongst women in low- and middle-income states [1]. Some of the challenges related to the management include late presentation, access to treatment and poor response to therapy [2,3]. Hypoxia is a common phenomenon linked to resistance to most forms of therapy in various malignancies including cervical cancer [4,5,6,7]. Hypoxia refers to a state of sub-physiological levels of tissue oxygen that develops due to excessive tumor growth which outgrows its blood supply and the inability of the new impaired blood vessels to keep up with the demand [8,9]. The presence or absence of hypoxia has therapeutic and prognostic implications.

Strategies to overcome hypoxia have been the subject of investigations over the years as treatment becomes more personalized. Several methods have been implemented to try and overcome hypoxia including intensity modulated radiation therapy (IMRT) which is closely linked to dose-painting, and hypoxia sensitizing drugs [10,11]. Due to the high costs and side effects of some of these therapies, it is vital to select patients that will likely benefit from these additional therapies. Therefore, detection of hypoxia in cancer lesions may help aid treatment planning.

The Eppendorf probe is considered the gold standard for hypoxia detection, however there are many reasons that have seen it fall out of favor. The analysis of genes and molecular markers that are upregulated in hypoxic states is another strategy to assess for hypoxia. These markers include hypoxia inducible factor 1 alpha (HIF-1α), vascular endothelial growth factor (VEGF) and carbonic anhydrase IX (CAIX), to name a few [12]. Other non-invasive methods have been sought and by far positron emission tomography (PET) imaging has been the most widely investigated. Fluorinated nitroimidazole derivates, specifically ^18^F-FMISO, were among the first tracers to be investigated for hypoxia imaging and are by far the most robust [13]. Due to its inherent limitations (slow tracer kinetics and non-specific washout) second and third generation nitroimidazole compounds such as ^18^F-FAZA, ^18^F-FETNIM and ^18^F-HX4 were developed [14,15,16]. The ^60/64^Cu-ATSM radionuclides were also developed and investigated as hypoxia markers [17]. The feasibility of all these tracers for hypoxia imaging in cervical cancers has been demonstrated, however some of them are not widely available. While imaging with the ^18^F-FDG plays a major role in oncology for staging and therapy planning, several studies have demonstrated that its role in imaging hypoxia is limited.

The chemistry of ^68^Ga makes for easy labelling with several peptides and molecules. With the increase in availability of the ^68^Ga generator this makes the ^68^Ga-labelled nitroimidazole derivatives attractive because ^68^Ga is available from a generator with a shelf life of almost a year [18]. The pre-clinical work on these tracers have demonstrated the feasibility of imaging hypoxia [19,20,21,22,23]. In these studies, ^68^Ga-Nitroimidazole compounds were more hydrophilic than the ^18^F-labelled radiotracers and were selectively taken up in hypoxic areas [24]. The aim of this paper was to investigate the feasibility of PET imaging of hypoxia with ^68^Ga-Nitroimidazole in cervical cancer lesions and to correlate imaging findings to findings on ^18^F-FDG PET/CT as well as immunohistochemical staining for HIF-1α.

## 2. Materials and Methods

### 2.1. Patients

Twenty women with histologically confirmed locally advanced cervical cancer were prospectively recruited into the study. The patients were enrolled consecutively from January 2020 to November 2021. The ^18^F-FDG PET/CT was performed as part of their work-up for initial staging to plan therapy. These patients were recruited as part of an ongoing study on hypoxia imaging. Informed consent was obtained from the patients for the scan as well as to access their hospital records. In the patients that were recruited, the hemoglobin levels at the time of imaging were determined and recorded. The study was approved by the Human Research Ethics Committee of the University of Pretoria (protocol number: 691/2019). All procedures were performed in accordance with the ethical standards of the institutional research committee in alignment with the 1964 Helsinki declaration and its latter amendments. 

### 2.2. Radiochemistry of ^68^Ga-Nitroimidazole

The synthesis of ^68^Ga-Nitroimidazole was performed as previously described [21]. Briefly, we obtained ^68^Ga from our inhouse ^68^Ge/^68^Ga generator (iThemba LABS, Somerset West, South Africa). We received the Nitroimidazole peptide complexed with TRAP chelator from Korea (Radiation Applied Life Sciences, Department of Nuclear Medicine and Cancer Research Institute of Radiation Medicine, Seoul National University College of Medicine, Seoul, Korea). This nitroimidazole residue, 2-(2-nitroimidazolyl)ethylamine was conjugated with TRAP via an acid-amine coupling reaction in dimethylsulfoxide (DMSO) using 2-(1*H*-benzotriazol-1-yl)-1,1,3,3-tetramethyluronium hexafluorophosphate (HBTU) as a coupling agent in the presence of *N*,*N*-diisopropylethylamine (DIPEA) as a base. Radiolabeling was conducted at pH of 4.5 at 95 °C in a heating block for 10 min. Labelling efficiency was verified by instant thin layer chromatography (ITLC) using 0.1 M Na_2_CO_3._ Radiochemical yields of the prepared derivatives were found to be >96% for all the derivatives. 

### 2.3. ^68^Ga-Nitroimidazole PET/CT Imaging Procedure

There was no specific patient preparation applied for the ^68^Ga-Nitroimidazole scan.

The injected activity of ^68^Ga-Nitroimidazole was weight based (1.8–2.2 MBq) and ranged between 111–185 MBq. We obtained a pelvic image at 30 min. post tracer injection. This was followed by a whole body (vertex to mid-thigh) PET/CT image at 60 min post tracer injection. Lasix was administered at the time of tracer injection. Patients were catheterized prior to the 60-min image to reduce bladder activity. PET imaging was acquired in 3D mode at 3 min per bed position. We used CT data for attenuation correction and for anatomic delineation of lesions. We performed image reconstruction using ordered subset expectation maximization iterative reconstruction algorithm (four iterations, eight subsets) followed by post reconstruction filtering with a Gaussian filter applied at 5.0 mm FWHM. 

### 2.4. ^18^F-FDG PET/CT Imaging Procedure

Patient preparation for ^18^F-FDG PET/CT included a minimum of 4 h of fasting which is in keeping with published guidelines [25,26]. Blood sugar before ^18^F-FDG injections was less than 7.1 mmol/L in all cases. The injected activity of ^18^F-FDG was between 3–5 MBq/kg. We imaged patients after an uptake period of 60 min. Thirteen of the patients were imaged on a Biograph 40 Truepoint PET/CT scanner (Siemens Medical Solutions, Lincolnshire, IL, USA), while the other seven were imaged on a Biograph Vision 450 PET/CT scanner (Siemens Medical Solutions, Lincolnshire, IL, USA). We performed a vertex to mid-thigh CT scan with parameters adjusted for patients’ weight (120 KeV, 40–150 mAs) with a section width of 5 mm and pitch of 0.8. 

### 2.5. Image Analysis

Two Nuclear Medicine Physicians with over two decades experience reporting PET/CT images reviewed the hypoxia ^68^Ga-Nitroimidazole PET/CT images. Reconstructed images were displayed as maximum intensity projection image, PET, CT, and fused PET/CT in the axial, coronal and sagittal planes on a dedicated workstation equipped with syngo software (Siemens Medical Solutions, Lincolnshire, IL, USA). 

### 2.6. Qualitative Analysis

The 60-min whole body images were analysed for bio-distribution as well as additional tumor related information. We performed qualitative assessment of the images and recorded our findings using a grading scale of 0–3 with 0 = no uptake/uptake less than normal background tissues, 1 = uptake similar to background activity, 2 = focal uptake above background activity and 3 = uptake markedly above background activity [27]. For this visual analysis, the background was considered as the blood pool. 

### 2.7. Semi-Quantitative Analysis

Semi-quantitative analyses were also used with calculation of SUVmax, SUVmean, HTV and Tumor to muscle ratio (TMR) as well as Tumor to blood ratio (TBR). The muscle tissue for the calculation of the TMR was the gluteus muscle and the aorta and left ventricle were used for the tumor to blood ratio. The tumor to muscle or blood ratio was calculated as the tumor SUVmax uptake divided by the SUVmean uptake in the gluteus muscle or the blood (aorta). Hypoxic tumors were identified based on the semi-quantitative analysis as those with at least one voxel with a ^68^Ga-Nitroimidazole signal greater than the calculated threshold. A semi-automatic spherical volume of interest (VOI) was drawn encircling the primary lesion in the cervix using an SUV threshold of 1.4 and a 3D isocontour of 41%. The VOI was manually adjusted to exclude areas of physiological uptake adjacent to the primary lesion. The maximum and mean standardized uptake values (SUVmax and SUVmean) and the hypoxic tumor volume (HTV) of the primary lesion in each patient were recorded. The hypoxic tumor volume (HTV) was defined as the volume in the ^68^Ga-Nitroimidazole PET dataset with an intensity greater than the determined threshold. This analysis was based on similar principles as the MTV determination. We divided the HTV by the MTV of the primary tumor to calculate a fractional HTV. 

^18^F-FDG PET/CT images were analyzed for the presence of nodal and distant metastases. Nodal metastasis was differentiated from inflammation based on the experience of the interpreting nuclear physicians using the following criteria as indicative of metastatic nodes: the presence of asymmetry, size, architecture or consistency (absence of fatty hilum/center, necrosis), the degree of uptake and the location. Disagreements were resolved by consensus. 

### 2.8. Immunohistochemical Assessment

The slides obtained from endocervical biopsies of the cervix for initial diagnosis were retrieved for immunohistochemical analysis. The slides were evaluated by an experienced pathologist from the department of Anatomical Pathology, National Health Laboratory Services (NHLS) at our institute. Hypoxia inducible factor 1 alpha (HIF-1α) monoclonal rabbit anti-human antibodies (clone EP118) were used to evaluate the HIF-1α expression. The tissue samples had all been fixed in 10% buffered formalin, processed and embedded in paraffin routinely. Sections were cut at 3 μm using a Leica TP1020 microtome and dried overnight at 60 °C. After deparaffinization in xylene, the sections were rehydrated in decreasing ethanol solutions and incubated in 0.3% hydrogen peroxide for 10 min, to block endogenous peroxidase. DAKO auto-stainer with PT link method of antigen retrieval was done. Citrate buffer solution (pH 6) for 20 min was done. Peroxidase was incubated for 5 min. Then, the slides were washed in PBS and put in the auto-stainer. Then the antibody was put in for 30 min and washed again in PBS. Envision fluid (polymer-peroxidase method, EnVision+/HRP, DAKO, Glostrup Kommune, Denmark) was added, followed by incubation for 30 min. Bound antibodies were visualized by using 0.05% 3,3′-diaminobenzidine solution (DAB solution, DAKO). Finally, sections were counterstained with Meyer’s hematoxylin and mounted in Entellan (Merck, Darmstadt, Germany). The grade of staining was defined via a light microscope. HIF-1α protein expression, (nuclear unless otherwise specified), was graded in intensity form 0 (negative) to 3 (strong), with 1 for weak intensity, 2 for moderate intensity. The distribution of staining was assessed as 25%, 50%; 75% and 100% of the tumor cells and allocated scores 1, 2, 3 and 4. A value of the sum of intensity and distribution out of 6 was used to score the positivity of the staining. HIF score = [intensity (I) + distribution (D)]. 

### 2.9. Statistical Analysis

Baseline clinical and demographic information of the patients was analyzed with descriptive statistics. Categorical data are presented as frequencies while continuous variables are presented as mean ± standard deviation (SD) or as median (interquartile range, IQR). Statistical analysis was performed using the commercially available software package SPSS 28.0. Normalcy was assessed by means of the Kolmogorov–Smirnov test. For non-normal distributed data, the Mann–Whitney test, Kruskal–Wallis test and Spearman-rank test were used when appropriate. For normal distributed data, ANOVA with post-hoc Bonferroni correction, the student *t*-tests and the Pearson correlation test were used when appropriate.

Multivariate analysis was performed using Cox-regression including in sequential order of statistical significance those variables that were found to be significant in univariate analysis. Statistical significance was defined as a *p*-value ≤ 0.05

## 3. Results

All 20 women included in the study underwent both ^68^Ga-Nitroimidazole PET/CT and ^18^F-FDG PET/CT on different days with a median interval of 6 days (range: 1–32) between the two scans. The mean age of the study population was 44.65 ± 11.43 years (range: 26–72). The majority (n = 16/20; 80%) of the patients had squamous cell carcinoma while the rest had various other histological subtypes of cervical cancer. Most patients were referred for a scan with an initial clinical impression of locally advanced disease, however, some of the patients were upstaged by the ^18^F-FDG PET/CT. Seventeen patients had a FIGO clinical stage of between IIB and IIIC2 while two patients had stage IVA and one stage IVB. Thirteen patients demonstrated pelvic lymph node metastases on ^18^F-FDG PET/CT. The median hemoglobin level was 10 g/dL (IQR: 9–11.75 g/dL). Demographic characteristics of the patients are displayed in Table 1 below.

### 3.1. Qualitative and Semiquantitative Analysis of the PET/CT Images

**Figure 1:**
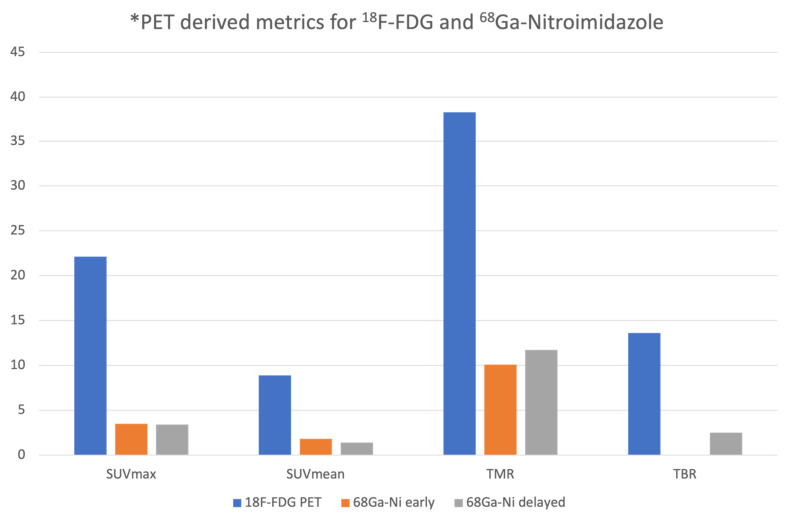
The mean SUVmax, SUVmean, tumor to muscle ratio (TMR) and tumor to blood ratio (TBR) for ^18^F-FDG and ^68^Ga-Nitroimidazole at 30 and 60 min. * PET: Positron Emission Tomography

The biodistribution was as anticipated with minimal tracer seen in the blood pool with excretion through the urinary system. A few patients demonstrated liver and gallbladder uptake. There was minimal background activity noted in the fat and muscles. The mean SUVmax and SUVmean of the primary tumor on ^18^F-FDG PET/CT imaging was 22.1 ± 21.4 and 8.9 ± 7.6, respectively; while the mean SUVmax and SUVmean of the primary lesion on ^68^Ga-Nitroimidazole PET/CT imaging was 3.54 ± 1.5 and 1.8 ± 0.8 for the early time point and 3.43 ± 1.5 and 1.4 ± 0.4 for the delayed imaging. There was no statistical difference noted in the mean SUVmax and SUVmean of the primary lesion between the two time points on the ^68^Ga-Nitroimidazole scan (*p* = 0.461; *p* = 0.510, respectively). Qualitatively, 12 patients had discernable focal uptake above background, three patients had uptake similar to background and the remaining five patients had no uptake on ^68^Ga-Nitroimidazole scan. The mean TMR on ^18^F-FDG PET/CT and at both time points on the ^68^Ga-Nitroimidazole scans were 38.3, 10.1 and 11.7, respectively. The mean TBRs on ^18^F-FDG PET/CT was 13.6 ± 11.3 and 13.5 ± 12.6 when using the aorta and ventricle as the blood pool surrogate. The median TBR on the delayed images on ^68^Ga-Nitroimidazole when using the aorta and ventricle were 2, while the mean TBR was 2.5 ± 1.6 and 3.3 ± 3.2. Figure 1 below demonstrates the semi-quantitative parameters on both ^18^F-FDG and ^68^Ga-Nitroimidazole PET/CT imaging.

The median MTV on the ^18^F-FDG PET/CT was 150.5 (18.41–548.93). The median hypoxic tumor volumes on the early and delayed images were 50 and 60.85, respectively. The hypoxic subvolume as a percentage of the MTV ranged from 2 to 98% (median 37%). 

### 3.2. Immunohistochemistry Findings

**Figure 2:**
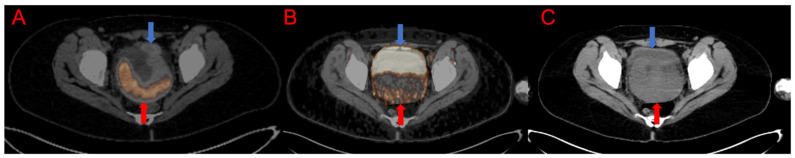
A 36-year-old female patient with FIGO stage II, squamous cell carcinoma of the cervix. (**A**). ^18^F-FDG PET transaxial image through the pelvis demonstrating uptake in the primary tumor (red arrow) with minimal uptake in the urinary bladder (blue arrow) (**B**). ^68^Ga-Nitroimidazole PET transaxial image through the same plane exhibiting spatially incongruent uptake with inhomogeneous uptake in parts of the tumor (red arrow) and intense activity in the urinary bladder (blue arrow). (**C**) is the corresponding CT only image in the same plane with target organs marked with arrows. The fractional hypoxic volume was 36%.

**Figure 3:**
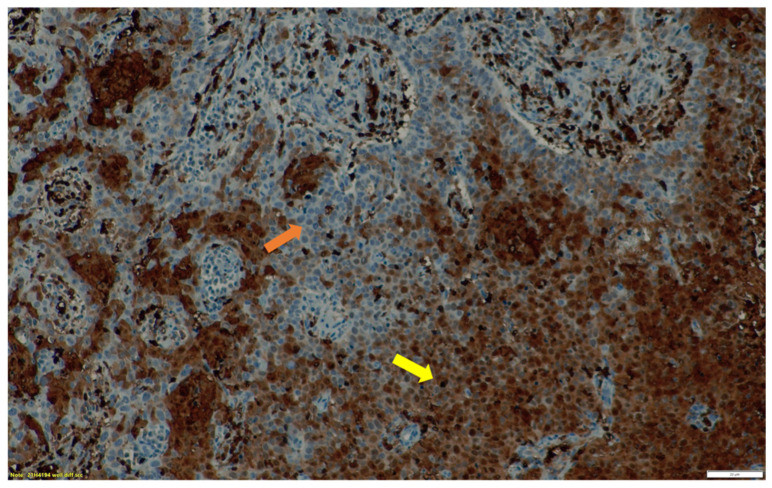
The immunohistochemical staining of HIF-1α expression in the endocervical biopsy specimen of the patient in Figure 2. Well differentiated squamous cell carcinoma ×20 magnification. The tumor areas with well differentiated cells (*yellow arrow*) with brown staining, demonstrate HIF-1α expression, while the areas with other cell types (*orange arrow)* demonstrate little to no HIF-1α expression.

We successfully retrieved histological specimen for 15 patients. Five patients had negative staining for HIF-1α on immunohistochemistry. The remainder of the patients had varying intensities of staining for HIF-1α with three (3) patients having weak staining, two (2) had moderate intensity and five (5) patients had strong intensity. When considering the hypoxia score, seven patients were considered positive for hypoxia. Table 2 represents the intensity and percentage distribution of the HIF-1α expression within the tumor specimen. In 70% (n = 7) of the patients with any level of HIF-1α expression on immunohistochemistry, we demonstrated uptake on the ^68^Ga-Nitroimidazole hypoxia PET scan (Figure 2 and Figure 3). Statistically there was a very weak, almost negligible correlation between these findings (r = 0.058; *p* = 0.837).

### 3.3. Correlation between ^68^Ga-Nitroimidazole PET/CT, ^18^F-FDG PET/CT Imaging and Immunohistochemistry

**Figure 4:**
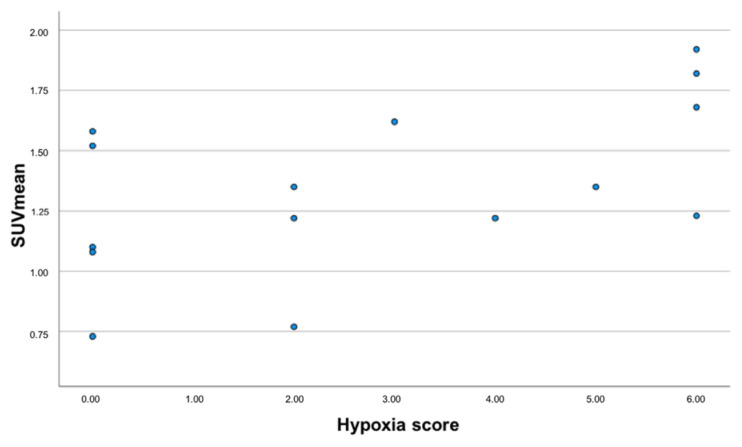
Scatterplot showing the relationship between the hypoxia score (data shown in the X-axis) and SUVmean of the primary tumor derived from the delayed ^68^Ga-Nitroimidazole PET images.

There was no statistically significant correlation between the hemoglobin levels, ^18^F-FDG SUVmax, ^68^Ga-Nitroimidazole imaging and immunohistochemical analysis. The median SUVmax and TMR on both ^18^F-FDG and ^68^Ga-Nitromimidazole at both time points did not demonstrate any statistical difference in hypoxic and non-hypoxic states as assessed by immunohistochemistry. When considering the hypoxia score, we found that five of the seven patients considered positive for hypoxia on immunohistochemistry, displayed uptake above background on the ^68^Ga-Nitroimidazole scan. Table 3. Demonstrates the results of the Kruskal–Wallis test assessing the difference between quantitative parameters on both ^18^F-FDG and ^68^Ga-Nitroimidazole PET/CT imaging in hypoxic and non-hypoxic states. There was no significant association between the presence of metastasis on ^18^F-FDG and the presence of hypoxia both on imaging and immunohistochemistry. None of the variables under study proved significantly different between those patients presenting with distant metastasis versus those who without (*p* ≥ 0.162). When assessing the HTV, we found no significant difference in the HTV of patients with positive results on immunohistochemical staining and those with negative results. Importantly we found a strong negative correlation between HTV and immunohistochemical staining (r = −0.660; *p* = 0.019). Additionally, hypoxia positivity proved moderately and significantly correlated to SUVmean values of the primary tumor derived from the early and delayed ^68^Ga-nitroimidazole PET examinations r = 0.531 (*p* = 0.42) and r = 0.580 (*p* = 0.024), see Figure 4. Additionally, upon dichotomization of the primary tumor hypoxia score (group 0, scores: 0, 1 and 2 and group 1, scores: 3, 4, 5 and 6) TMR derived from the early ^68^Ga-nitroimidazole PET examination proved significantly higher in tumors with score 1 versus those with score 0 (13.2 ± 7.3 versus 8 ± 13.2, respectively). 

## 4. Discussion

The presence of hypoxia in solid tumors has a bearing on treatment and outcomes; therefore, non-invasive modalities that can map it are essential for therapy planning. Several PET tracers have been utilized for this purpose in various cancer entities with variable results. Our study aimed to image hypoxia in cervical cancer lesions/tumors with ^68^Ga-Nitroimidazole which is more hydrophilic and has demonstrated improved properties compared to 1st, 2nd and 3rd generation ^18^F-labelled nitroimidazole tracers. The biodistribution was as expected with intense activity seen in the kidney and urinary bladder because of the hydrophilicity. The qualitative assessment revealed that two-thirds of the patients had uptake in the primary tumor above background. This is similar to the >50% rate of hypoxic tumors in studies using invasive needle electrode measurements [6,7,28]. In a study of 38 women, Dehdashti and colleagues found discernable ^60^Cu-ATSM uptake in all but one patient [29]. Another study using ^18^F-FMISO also detected focal areas of uptake in all 16 patients [30]. Although the patient population in both studies was similar to ours (locally advanced tumors), the differences seen in the numbers of patients with uptake may be related to the differences in the tracers and tracer kinetics as well as interpretation criteria. Most studies on hypoxia imaging (pre-clinical and clinical) report on the SUVmean or none at all and we found the SUVmean on the delayed ^68^Ga-Nitroimidazole to be strongly correlated to the positivity on immunohistochemical staining. This finding is most likely related to the heterogeneity of hypoxic regions within a tumor and may explain why most papers on this subject have reported on the SUVmean as opposed to the SUVmax. The median SUVmax in our series was 3.63 and 3.27 at 30 and 60 min, respectively, which is comparable to that reported in the clinical work using ^18^F-FMISO in cervical cancer patients [30]. The other parameter that has been more commonly reported on, in the context of hypoxia imaging using different tracers is the TMR or TBR. Preclinical studies using ^68^Ga-labelled nitromidazoles have demonstrated that TMR and TBR were highest at 1 h post imaging. We confirmed this finding as we found a median TMR of 8 ± 4.8 and 10 ± 5.1 at 30- and 60-min post injection, respectively. Initial pre-clinical work on ^68^Ga-Nitroimidazole revealed median TMRs as high as 7.41 ±1.12, 5.70 ± 2.5, 5.64 ± 0.8 [19,20,21]. The work on ^64^Cu-ATSM also revealed TMR 7.3 ± 1.8 [31]. These high TMRs contrast with the TMRs when using ^18^F-labelled tracers and this may be attributed physical properties of these tracers which result in faster clearance from background tissues. We found no significant difference in the TMR and TBR between the 30 min and 60 min time points, suggesting that images may be obtained as early as 30 min post tracer injection without significantly compromising the image quality. Interestingly we also found higher TMRs on the early ^68^Ga-Nitroimidazole scan in patients with hypoxia on immunohistochemistry than those with no hypoxia. This may further support early imaging. This is in contrast to ^18^F-FMISO which has prolonged imaging times up to 2 h post tracer injection.

There is an abundance of literature demonstrating hypoxia in cervical cancer lesions using ^18^F-labelled tracers and ^60/64^Cu-ATSM, however very few of them correlated their findings to immunohistochemistry which may be considered the gold standard. There are various genes that are upregulated in hypoxic environments including vascular endothelial growth factor (VEGF), Carbonic anhydrase IX (CAIX) and osteopontin to name a few. In a study of 44 women with advanced cervical cancer, the authors found no correlation between the expression of HIF-1α and tumor oxygenation as detected by an Eppendorf device [32]. Vercellino and colleagues assessed the feasibility of imaging hypoxic lesions with ^18^F-FETNIM and correlated their findings to osteopontin which is also upregulated in hypoxic environments. They found no correlation between levels of osteopontin and ^18^F-FETNIM uptake. Similarly, we also failed to find a correlation between the HIF-1α expression on immunohistochemistry and ^68^Ga-Nitroimidazole imaging. We found that almost half of the patients with any level of HIF-1α expression on immunohistochemistry had discernable hypoxia on PET imaging, while five of the seven patients with positive hypoxia scores had uptake on ^68^Ga-Nitroimidazole PET. This is contrary to the study by Grigsby et al. who correlated VEGF, CAIX, cyclo-oxygenase-2 (COX-2), epidermal growth factor and apoptotic index with ^60^Cu-ATSM PET imaging of tumor hypoxia. Immunohistochemical markers were expressed in most, if not all the tumors seen to be hypoxic on ^60^Cu-ATSM PET imaging [33]. Immunohistochemical analyses are fraught with challenges including operator dependence and sampling issues etc., and this may be a reason for the lack of correlation of these findings with imaging as seen in our study.

We believe there are unique patient groups or outcomes that deserve a special mention because of their interesting or unexpected findings. Two patients, namely patient 4 and 5 (Table 2) demonstrated hypoxia on immunohistochemistry, however their ^68^Ga-Nitroimidazole PET scans demonstrated no discernable areas of uptake. The reasons for the above may be related to size of the tumor which may render the lesion/s prone to partial volume effect or being missed because of the inherent resolution limits of the imaging unit. In three patients (7, 9 and 13), we note the reverse, wherein the despite lack of HIF-1α, the ^68^Ga-Nitroimidazole PET scan demonstrated uptake above background tissues. This outcome may be related to sampling errors for the HIF-1α staining in view of the heterogeneous nature of hypoxia. 

Most oncology guidelines recommend the use of ^18^F-FDG PET/CT in the staging and restaging of most malignancies including cancer of the cervix [34]. Often, ^18^F-FDG PET/CT images are used for the visual estimation of uptake of hypoxia tracers within tumor lesions. Furthermore, in-vitro studies have demonstrated that hypoxia results in increased FDG uptake [35,36,37]. There are several studies which have correlated the findings on hypoxia PET imaging with those from metabolic imaging. Grigsby et al. found no correlation between uptake parameters on ^18^F-FDG and ^60^Cu-ATSM PET scans, however they did note a significant correlation between the presence of ^18^F-FDG positive lymph nodes and the findings on ^60^Cu-ATSM PET [33]. The lack of correlation between the metabolic and hypoxia imaging has been also demonstrated in other tumor entities [38,39]. Similarly, we could not demonstrate any correlation between PET-derived parameters (SUVmax, SUVmean, TMR and TBR) on the metabolic and hypoxic imaging. We also found no correlation between the presence of any metastasis on ^18^F-FDG PET and ^68^Ga-Nitroimidazole. The hypoxic volume was always less than the metabolic tumor volume in our patient cohort. This is in support of the reports of heterogeneity of hypoxia, therefore further highlighting the importance of hypoxia mapping with imaging studies. 

We found a significant strong negative correlation between HTV and immunohistochemistry (r = −0.660; *p* = 0.019). This finding was not anticipated. We postulate that this finding may be due to several factors including tumoral environmental issues and technical factors. We believe that other factors besides upregulation of HIF-1α are at play in the hypoxic environment. A possible theory is the presence of a feedback mechanism between markers/genes within the tumor and HIF-1α. Another plausible explanation may be related to the different isoforms of HIF-α, as it has been shown that there are at least three, namely HIF-1α, HIF-2α and HIF-3α [40]. These α-subunits are regulated at the protein level by oxygen dependent mechanisms. It has been shown that HIF-1α expression is higher in acute hypoxia whereas that of HIF-2α is higher in chronic hypoxia [41,42]. This finding calls for larger studies with modified and improved protocols to be conducted as to unravel the association we found. 

To the best of our knowledge, this is the first study to assess this novel ^68^Ga-Nitroimidazole tracer in patients with cervical cancer. This very fact may prove to be a limitation since there are no prior studies to compare our findings to, therefore most of our comparisons are with pre-clinical studies, other ^18^F-labelled or the ^64^Cu-labelled hypoxia tracers. We could not draw any strong conclusion because of the small sample size. The main excretory pathway of the more hydrophilic ^68^Ga-Nitroimidazole is through the renal system. This resulted in intense bladder uptake that may pose as a challenge for pelvic malignancies. Despite urinary catheterization of the majority of our patients, the urinary bladder was often incompletely drained, and this may pose a challenge in the interpretation of the scan. Therefore, for pelvic malignancies, a tracer with minimal renal excretion or the employment of radiomics, may optimize the interpretation of the findings. The immunohistochemistry was performed on biopsy specimen that were collected at initial diagnosis and this may misrepresent the true status of hypoxia in the entire tumor lesion. Lastly, immunohistochemical analysis is highly operator dependent and this too may pose as a limitation in our study. Future studies with pre-operative patients that will undergo surgery and subsequent immunohistochemistry on more representative samples of the surgical specimen may yield improved results. While it was our desire to perform survival analysis, local factors e.g., lack of centralized health informatic systems, proved to be a challenge. This information may have added further insight in this area.

## 5. Conclusions

We found that there was no difference in the tumor uptake on ^68^Ga-Nitroimidazole PET/CT between early (30 min) and delayed (60 min) imaging, which may suggest that imaging can be acquired early without compromising the tumor to background ratio. Although we detected hypoxia in two-thirds of the patients on ^68^Ga-Nitroimidazole PET imaging, we found no significant relationship between HIF-1α and clinicopathological features or ^18^F-FDG and ^68^Ga-Nitrimidazole PET/CT parameters. However, we found higher TMRs and SUVmeans in patients with hypoxia as assessed on immunohistochemistry. Furthermore, there was a negative correlation between HTV as assessed by PET hypoxia imaging and HIF-1α staining. Further, larger studies are required to determine the prognostic value of using ^68^Ga-Nitroimidazole PET imaging to predict the pathological and prognostic course of cervical cancer.

## Figures and Tables

**Table 1:** Demographic characteristics of the patient cohort.

Variable	Frequency	Percent
**Age (years)**		
Mean ± SD	44.65 ± 11.43	
Range	26–72	
**Histological subtype**		
Mucinous endocervical carcinoma	1	5.0
Papillary squamourethelial carcinoma	1	5.0
Squamous cell carcinoma	16	80.0
Papillary surface serous carcinoma	1	5.0
Adenocarcinoma	1	5.0
**FIGO stage**		
IIB	3	15.0
IIIA	1	5.0
IIIB	9	45.0
IIIC	2	10.0
IIIC1	1	5.0
IIIC2	1	5.0
IVA	2	10.0
IVB	1	5.0

SD: Standard Deviation; FIGO: International Federation of Gynecology and Obstetrics.

**Table 2:** Immunohistochemical analysis and correlation to ^68^Ga-Nitroimidazole imaging qualitative assessment.

Patient No.	Histological Subtype	Intensity of Staining of HIF-1α	Percentage Distribution of HIF-1α Expression	Sum of Intensity and Distribution (Out of 6)	Qualitative ^68^Ga-Nitroimidazole PET Findings
***1***	Squamous cell carcinoma	3	25%	4	2
***2***	Squamous cell carcinoma	3	75 %	6	2
***3***	Squamous cell carcinoma	3	75%	6	2
***4***	Papillary surface serous carcinoma	2	75%	5	0
***5***	Squamous cell carcinoma	3	75%	6	0
***6***	Adenocarcinoma	3	75%	6	2
***7***	Squamous cell carcinoma	0	-	0	2
***8***	Squamous cell carcinoma	2	25%	3	2
***9***	Squamous cell carcinoma	0	-	0	2
***10***	Papillary squamo-urothelial carcinoma	1	25%	2	1
***11***	Squamous cell carcinoma	0	-	0	1
***12***	Squamous cell carcinoma	1	25%	2	2
***13***	Squamous cell carcinoma	0	-	0	2
***14***	Squamous cell carcinoma	0	-	0	0
***15***	Squamous cell carcinoma	1	25%	2	2

Intensity of staining: 0 = no staining, 1 = weak intensity, 2 = moderate intensity, 3 = strong intensity. Sum of intensity and percentage distribution out of a total of 6 (see methods section for detailed description). Values above 3 were considered positive for hypoxia. Qualitative ^68^Ga-Nitroimidazole PET findings: 0 = no uptake, 1 = uptake similar to background, 2 = focal uptake above background, 3 = focal uptake markedly above background.

**Table 3:** SUVmax and TMR from the ^18^F-FDG and ^68^Ga-Nitroimidazole PET/CT scans correlated with the HIF-1α expression.

	Hypoxia on Staining					
	Negative	Weak	Moderate	Strong		
	Median (Range)	Median (Range)	Median (Range)	Median (Range)	K	*p* Value
^18^F-FDG SUVmax	17.32 (11.82–17.81)	21.21 (11.64–22.42)	13.70 (13.41–13.99)	17.50 (16.09–30.77)	2.927	0.403
^18^F-FDG TMR	30.00 (21.00–43.00)	38.00 (22.00–46.00)	27.00 (27.00–27.00)	32.00 (18.00–50.00)	0.670	0.880
^68^Ga-Ni SUVmax(1)	3.73 (1.00–4.00)	3.38 (3.00–4.00)	2.08 (2.00–2.00)	4.10 (2.00–5.00)	2.795	0.424
^68^Ga-Ni SUVmax(2)	3.71 (1.60–4.19)	3.06 (3.04–3.15)	1.77 (1.62–1.91)	3.57 (2.78–4.32)	4.342	0.227
^68^Ga-Ni TMR	8.00 (4.00–10.00)	9.50 (8.00–11.00)	10.00 (6.00–14.00)	15.00 (7.00–24.00)	2.669	0.446
^68^Ga-Ni TMR 2	11.00 (6.00–16.00)	15.00 (7.00–17.00)	7.50 (7.00–8.00)	12.00 (7.00–26.00)	1.852	0.604

K: Kruskal–Wallis test, ^68^Ga-Nitroimidazole maximum standardized uptake value (^68^Ga-Ni SUVmax): ^68^Ga-Ni SUVmax (1) and ^68^Ga-Ni SUVmax (2): SUVmax at 30 and 60 min post tracer injection, respectively; ^68^Ga-Nitroimidazole tumor to muscle ratio (^68^Ga-Ni TMR): ^68^Ga-Ni TMR and ^68^Ga-Ni TMR 2 are the tumor to muscle ratio at 30 and 60 min post tracer injection, respectively.

## Data Availability

The data presented in this study are available on request from the corresponding author.
